# Application Research of Public Art in the Design of Old People's Rehabilitation Space Based on Virtual Information Interaction Platform From the Perspective of Developing Economies

**DOI:** 10.3389/fpubh.2022.917330

**Published:** 2022-05-27

**Authors:** Shaoqing Zhang

**Affiliations:** College of Landscape Architecture and Art, Henan Agricultural University, Zhengzhou, China

**Keywords:** old-age, recuperation space, virtual information interaction platform, public art, design

## Abstract

To solve the problem of the design of the old people's recuperation space, the virtual information interaction platform is used to study the public art application in the design of the old people's recuperation space. Firstly, the principles of interactive design are expounded, and secondly, the existing institutions for the old people are investigated. Under the premise of optimizing the functions of the facilities, the concepts of humanistic care, emotional care and humanization in public art are integrated into the design of the old people's rehabilitation space, to solve the long-term negative impression of the old people's repression and indifference to the old people's care institutions. The construction of the scene allows the old people to experience some operations with the help of the virtual information interaction platform. In the modern elderly rehabilitation space, the attention and application of public art design will inevitably bring spiritual and material help to the old people in their later years, and create a happy, peaceful, and comfortable elderly life for them. The survey results manifest that 65.3% of urban old people and 71.8% of rural old people feel that they cannot keep up with the pace of development. Through the analysis and discussion of the physiological and psychological characteristics of the old people, the whole survey denotes that the physiological functions of the old people are declining, which seriously affects their normal life. Therefore, the design of the rehabilitation space for the old people should not only meet the basic needs of life, but also analyze the space design from the perspective of humanization and emotion. An ecological, natural, and human settlement environment has been established. The recuperation space is designed for the needs of different old people, which helps the old people to eliminate loneliness, enhance their value of the old people, and make life full of joy and meaning for the old people.

## Introduction

The aging of the population is gradually becoming a worldwide topic and an important issue for many countries at this stage. Since the end of the last century, China has entered an aging society. China is a country with a large number of old people population in the world, and also one of the countries with the fastest growing degree of aging (1, 2). How to deal with the needs of many old people and provide more comfortable living conditions for older people is one of China's current national conditions. From the change of social and family roles, the old people are prone to psychological imbalance, which leads to loneliness. In addition to caring for their physical health, the focus of the old people's emotional space is equally critical, so that their later life can be full of happiness and care. In China, with the rapid growth of the elderly population and a large base, little attention has been paid to the old people, and less care has been given to the old people (3). After the old people withdrew from the social role, most of the time they lived at home or in the old-age institutions, and they were more dependent on their living space in their later years. They need to go to public life and communicate with others. Therefore, the living space of the old people should create a living space suitable for health, recreation, emotional communication, social play, and pleasing life (4).

There are many studies on the old people. For example, Lee et al. (5) developed eight personas representing four different user groups in the context of home appliance use: visually impaired, hearing impaired, spinal cord impaired, and the old people. Each has a persona as a tool to understand the target user, a persona card representing their task obstacles, frustrations, needs and references, and a cartoonish persona. In this study, two common accessibility issues and two role-specific issues are addressed in each user group (5). These questions are presented in the language roles of stakeholders to help them understand users. Tsuchiya et al. (6) conducted a study on the evaluation of smart home control systems for the old people with a sample of 10 users in inland cities in Brazil. The control system consisted of a prototype using a web-based mobile application, which was developed taking into account requirements obtained from previous studies and recommendations from the literature The study demonstrated clearly the importance of providing information directly by identifying the user's home location in the application, not hiding information under scrolling, appropriately using images and videos in the system to avoid confusion, limiting the number of open windows to maintain context, avoiding unclear interactive elements to facilitate, and showing the visuals of the room and appliances on the screen (6). The findings could help improve the old people's interactions with smart home systems, especially in rural areas of developing countries. And public art is no longer an unfamiliar word in contemporary China. After the reform and opening up in the 20th century, it became a topic of great concern to Chinese artists in the 1990s (7). The development of contemporary Chinese public art is gradually developed and improved along with the adjustment of national policies, the marketization and internationalization of the economy, the socialization of rapid urban development, and the continuous improvement of citizens' rights. Since the beginning of the 21st century, public art has shifted from intense topic discussion to practical research in China, and is entering the urgent needs and rapid development of contemporary society (8, 9). With the rapid development of China's economy and the urgent need for public art culture, China's contemporary public art and its theories are constantly advancing and exploring, and are influenced by relevant external experience and theoretical research. Combined with the current situation of Chinese society and the needs and problems of public art culture development, corresponding public art practices and theories have gradually been produced (10). The main feature of its theoretical research is to gradually shift from the study of the external form and style of public art and the application of urban public space to the study of public art and sociology. The study of the simple aesthetic value orientation of public art has been transformed into the study of the relationship between public art and urban subject culture and citizens' daily public life. Contemporary Chinese public art no longer exists in only one form of art. Nowadays, the publicity and public participation of public art are emphasized. Various services are provided to the public and diverse forms of social public space, which is the overall social and cultural mission. At present, it pays more attention to various vulnerable groups in the society and realizes social fairness and democratic rights in the form of art (11). Therefore, based on Chinese contemporary public art, the recuperation space for the old people is studied.

With the improvement of people's living standards, its actual functions should be fully considered in the design, to meet the needs of the old people for material life as much as possible. According to the survey, it is found that most of the old people lack communication, have a sense of distance, and lack communication bonds with each other. Through the reasonable planning of public space, it is the main motivation of this research to make the people in the space connect with each other and the environment, and make more contacts. The old people have many needs on the spiritual level, which can be roughly divided into spiritual needs such as emotional, social, entertainment and self-realization. Today, the existing old-age care places in the society can no longer fully meet the spiritual needs of the old people, and the design also needs to be improved. The main innovation is to solve the spiritual needs of the old people. It is designed from three aspects: social, participatory, and service-oriented. Interactivity is applied to the layout of the public space of the old people apartment to solve the needs of the old people. This research explores how to carry out the interactive design in the design of rehabilitation space, enhance the ability of the old people's care services, improve the service level for the old people, and enrich the theory of social elderly care, which has good research value.

## Methodology

### The Outdoor Environment Design of the Rehabilitation Space for the Old People

The outdoor environmental space refers to the spatial extent of the specific area except the interior of the building, which is different from the open space of the city (12). The old people in nursing homes have different cultural levels and different physical qualities. Therefore, in order to meet these different needs of the old people, the way is also ever-changing (13). The overall differences in the activities of different types of elderly groups and the different characteristics of their activities have become the basis for the outdoor environment design in the elderly rehabilitation space.

Necessity, spontaneity, and sociality are several environmental categories in the aged rehabilitation space. The main activities in the entire elderly rehabilitation space are mainly based on essential and spontaneous activities. All activities of the old people are particularly dependent on the quality of the environment and space. When the quality of external activity space is relatively bad, old people mainly focuses on activities that are essential to life, while other interactions are basically not going on. However, when the quality of the activity space environment is good, they will spontaneously generate diverse activities, and social activities will increase.

When setting up the activity space, special consideration is given to the physiological characteristics of the old people. Ventilation and lighting must be very good. However, the outdoor activities of the old people cannot be located at the vents, and the outdoor rest points for the old people should be continuous. The old people have limited physical strength and the outdoor seating should be installed along the road.

**Figure 1:**
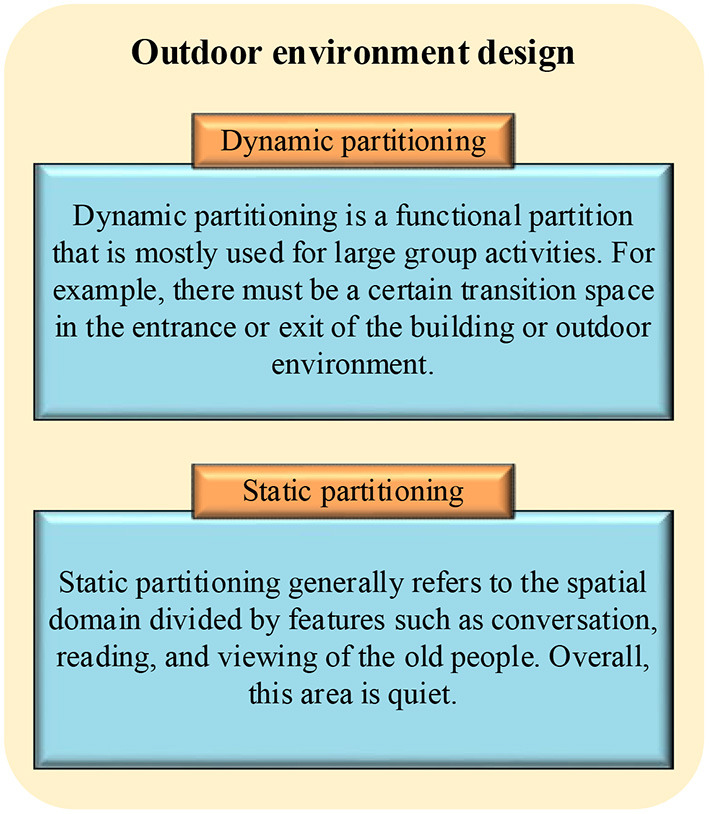
The outdoor environment design of the elderly rehabilitation space.

The outdoor environment design of the rehabilitation space for the old people is divided into two parts, as shown in Figure 1. Outdoor environment design is usually divided into dynamic partitioning and static partitioning. Static partitioning generally refers to the spatial domain divided by features such as conversation, reading, and viewing of the old people. Overall, this area is quiet. Dynamic partitioning is a functional partition that is mostly used for large group activities. For example, there must be a certain transition space in the entrance or exit of the building or outdoor environment. Seats and ornamentals for leisure must be provided. The composition and diversity of space is emphasized when designing outdoor spaces. Light, shadow, and visual effects should be appropriate for older people's life requirements, which avoids the overly shocking picture and space composition.

### Functional Composition of the Elderly Rehabilitation Space

**Figure 2:**
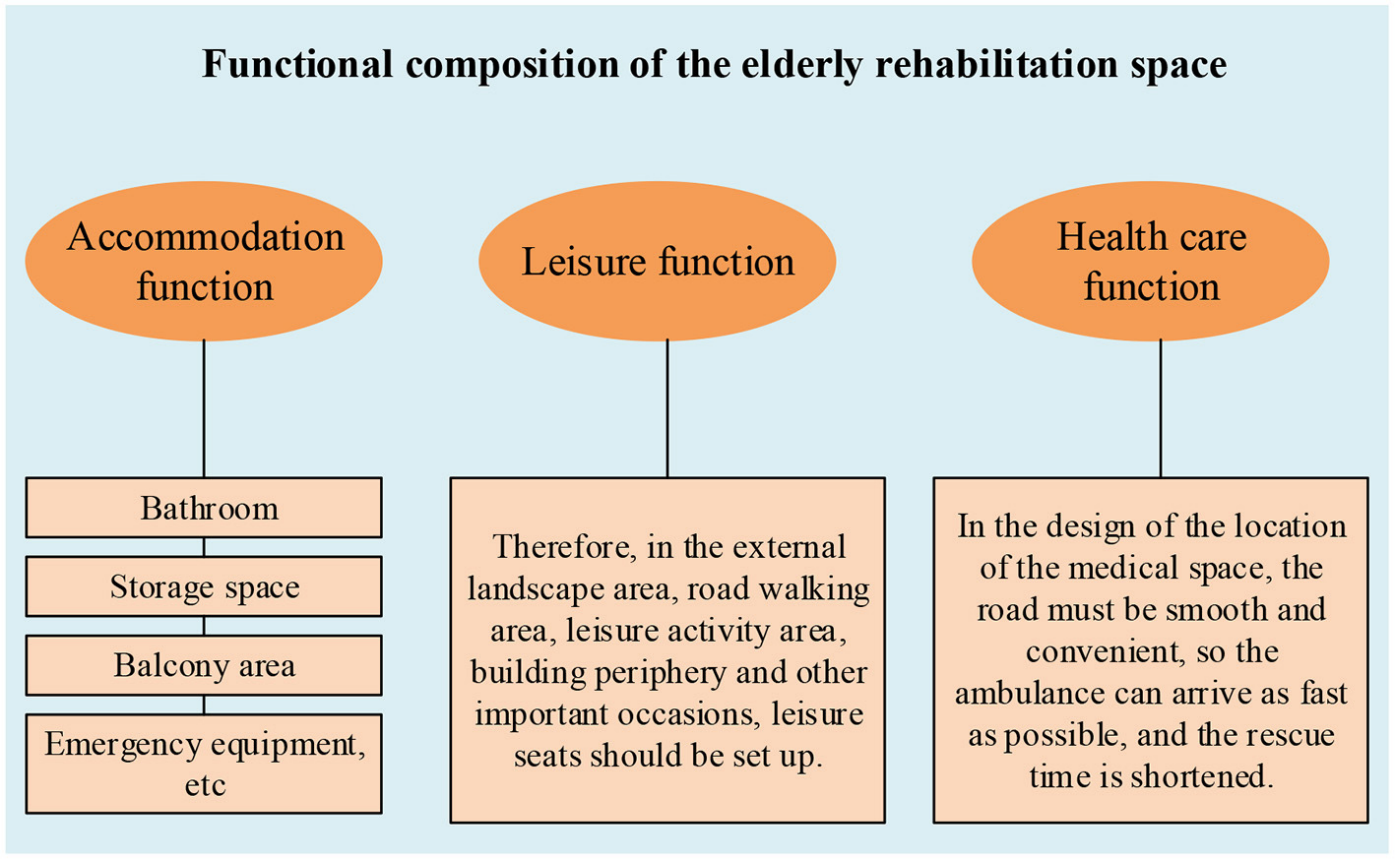
The functional composition of the elderly rehabilitation space.

The functional composition of the elderly rehabilitation space is shown in Figure 2.

#### Accommodation Function

The accommodation function is the most important requirement of the old people in the rehabilitation space. Old people generally spend most of their time in the living space. Therefore, the accommodation environment for the old people must be taken very seriously. According to the physiological characteristics of the old people and the requirements of life use, the necessary functional facilities, such as the bathroom, storage space, balcony area, emergency equipment, etc., should be considered in the design. At the same time, privacy requirements should also be considered.

#### Leisure Function

In rehabilitation space, the problem of rest must be involved, whether indoor or outdoor. In the discussion of rehabilitation space, the external environment is the main design object. Therefore, in the environment of external communication activities, reasonable rest places must be set up. Old people often need to sit and rest during outdoor activities for physical reasons. Therefore, in the external landscape area, road walking area, leisure activity area, building periphery and other important occasions, leisure seats should be set up, and the number is higher than the design ratio in the city square. At the same time, the ergonomic requirements of the old people should be met.

#### Health Care Function

Old people's physical condition is much worse than before, especially the elderly people. Sudden infirmities of age often occur and emergency medical care is needed. Therefore, in the rehabilitation space, the design of the medical function is very important. This guarantees the safety of the old people in the event of an emergency. In the design of the location of the medical space, the road must be smooth and convenient, so the ambulance can arrive as fast as possible, and the rescue time is shortened (14).

### Barrier-Free Design Requirements

Barrier-free design is an important factor that must be considered and embodied in rehabilitation space for the elderly. Barrier-free design helps old people perform normal activities. This assists to solve the problems of old people, such as the decline of physiological function and inconvenience of movement. Barrier-free design should be strictly observed in all parts of rehabilitation space for the elderly and in the placement of functional facilities (15).

#### The Influence of Old People's Physical Decline

Design of the ramp: In the rehabilitation space of the elderly, it is inevitable that there will be road ramps, including stairs with a ground height difference environment. Therefore, the design of the ramp should be based on gentle slopes as much as possible. It is convenient for wheelchairs and other equipment to walk, and must be equipped with safety rails next to it. In addition, there should be reasonable ramps side by side in places where there are steps.

#### Use of Non-slip Materials

The balance ability of the old people and the flexibility of the limbs are bad, so the floor tiles must be strictly controlled. Floor coverings with high surface smoothness are strictly prohibited, which makes it easy for the elderly to slip. Especially in corridors, stairs, sanitary napkins, etc., these are places where old people are prone to accidents. In outdoor activities, paving should be based on flat and non-slip materials.

#### The Influence of Hearing and Vision of Old People

After the decline of physiological function, the first manifestation is the change of hearing and vision. Therefore, in the maintenance of the modern elderly, in view of the hearing loss of the old people, there are different amplification devices in different space areas to ensure the normal activities and exchanges of the elderly. For the decline of vision, the following aspects are considered.

Eye-catching signs are set on the road and orientation search, and distinguished by color and shape. Good lighting systems are equipped to assist in lighting. Through voice control and other means, these lighting devices are connected, especially at night. Intelligent devices, some acousto-optic signal devices, such as telephones, doorbells, elevator stop signals, call service facilities, etc. should be strictly observed in the design of the indicator lights. This is brighter than the usual general equipment and is clearly legible for identification. The intelligent guiding device is configured to be carried by the elderly. Whether indoors or outdoors, the location of the elderly can be searched at any time to guide the direction, which helps the visually obsessed old people to live comfortably.

#### Management Discussion

A sound management system for the elderly rehabilitation space was established. The professional knowledge and skills training of nursing staff was strengthened and the quality of personnel services was improved (16). Moreover, the state should have a strong and sound legal system supervision. The healthy life of the old people is advocated. Self-care ability is improved through the efforts and exercise of the old people themselves. Helping the old people to rebuild their confidence is also one of the important purposes of the construction of the elderly rehabilitation institutions.

### Physiological Characteristics of the Elderly and Its Impact on Public Art Design in the Rehabilitation Space of the Elderly

The physiological function of the human body will continue to decline, which is the objective law of human life development. However, the decline of physiological functions will directly affect the lives of the old people and their ability to adapt to the natural environment and living environment. In addition, there will be a significant decline in communication skills. Therefore, in the design of the rehabilitation space for the elderly, the design of the living space and the outdoor environmental landscape should be based on the actual situation of the elderly.

The process of the old people's decline is divided according to the “old people's residential building design guidelines”, which are mainly in four stages: The healthy functioning period of the body function is 60–64 years old. The independent period of self-care is 65–74 years old. The inconvenience of action is 75–84 years old. 85 years of age or older is a serious debilitation and is listed as a key nursing period.

The normal growth and development of the human body will change over time. As the age increases, the overall signs of physiologic weakness are more pronounced. The growing age has gradually degraded the functions of the various parts of the old people's body. Especially after 70 years of age, the signs of this decline are more obvious, which will seriously affect the normal physiological function of the old people. As physiology decreases, the design tools and methods used for the needs of the old people will change. More requirements are also placed on the design of public art in the aged rehabilitation space. According to the special requirements of the old people's physiological functions, public environmental facilities or public works of art that meet the overall requirements are designed.

The degree of self-care of the old people directly determines the degree of privacy of the entire living space and the level of living services and care that the maintenance center needs to provide to the old people. Under normal circumstances, the continuous decline of the old people's self-care ability will directly lead to the weakening of the privacy of the entire old people's living environment. At the same time, the maintenance center will be required to strengthen the care and companionship of the old people who cannot take care of themselves. The entire care phase of an old person consists of the following steps: life self-help period; partial care period; comprehensive care period; termination period. From self-help to termination, the life of the old people is an unchangeable trend. The level of care varies according to the difference in care required at each stage of human normal aging.

In combination with the differences in the physiological characteristics of the older people mentioned above, there is a big difference between adults and old people. The physiology of older elderly people is more fragile. Therefore, more care is needed for older people, considering the particularities of life of older people. Thus, there are several factors that must be considered when designing the old people rehabilitation space.

#### Auditory System Design

A very prominent feature of the old people group is the sharp deterioration of hearing due to the decline of physiological functions. In the investigation of old people rehabilitation institutions, there have been many cases in which the old people have difficulty communicating due to severe hearing loss. Old people are eager to have sensitive ears to get useful information in life. A sharp decline in hearing can significantly reduce the enthusiasm of older people to participate in activities. Older people will have obvious conversational conflicts in their hearts, and they need tools to help their ears get information.

However, when the old people are alone, they like quiet places and spaces very much. They don't want their quiet state to be disturbed by loud noises. In a survey of nursing homes, it was found that old people were disturbed by noisy external voices because it lived close to the main road. Therefore, for the phenomenon of hearing loss of the old people, the design of the elderly rehabilitation space also brings a lot of challenges.

#### Vision System Design

A decrease in vision is an important manifestation of the decline in the physiological function of the old people. In previous interviews with several elderly institutions, it was learned that many problems, such as dazzling eyes and difficulty in light perception, will arise because of the impaired vision. The old people went in the wrong direction and even stumbled because they could not see the marker. Therefore, in the design of the elderly rehabilitation space, the visual situation of the old people must be carefully understood. The subject buildings, road-pointing signs, and indexing of important places involve visual observations of the old people, and any environment must be treated with rigorous care. For example, a very prominent reminder sign needs to be placed on the floor building, the stairway, the corner, the slope, and so on.

#### Tactile System Design

Old people's tactile system is also declining with age. They rely on their physical touch to feel the environment and place very high demands on the environment. For cold and hot, the old people are significantly less able to accept the environment than young people. For example, the old people like to sit around the fire source in winter, or need heating facilities. In summer, they need a cool and ventilated environment. Therefore, there are many requirements for the design of the old people rehabilitation space, which is different from the treatment of young people. At present, the design of old-age maintenance institutions is seriously inadequate. In the future, the elderly rehabilitation institutions have higher requirements.

#### Barrier Free Design

The concept of barrier-free design must be strictly enforced throughout the design of the elderly rehabilitation space. The old people will continue to decline in their physiological functions, their physical activities will be inconvenient, and the ability to adapt to the environment will show significant decline. Steps, stairs, corridors, handrails, toilets and other very common public facilities, young people usually do not pay much attention to. However, as the physical and mental functions continue to decline, the old people will have inexplicable fears and potential threats to these common public facilities. In the course of practical research, it was found that most of the old people needed crutches and other supporting tools in their activities.

#### Activity Space Design

As a result of the comprehensive analysis of the physiological characteristics of the old people, as their age increases, the functional organs will also weaken. The overall health status will be significantly lower than in the past. Therefore, the old people will have a very personal health status in their lives, and they are also very concerned about the disease.

From the analysis of physiological characteristics, the old people need a public green space with good environmental quality to breathe fresh air and exercise. In addition, the old people have come out of the life stage of youth and live in society. However, they have withdrawn from many social things such as social interaction. Second, the dominant role of the old people in society is also changing anyway. From the collective work of the unit to the individual work in the family, the entire social environment and social group relations are also changing. Children lived in other places all the year round and stayed away from the elderly. The lively atmosphere of the family had changed. Therefore, the new living environment, life progress and lifestyle will bring a sense of discomfort. These inevitably lead to the feeling of anxiety and loneliness in the old people. Compared with some families with superior economic conditions, the high-cultural and healthy old people will have great enthusiasm and interest in social, group entertainment and social interaction. This type of old people is hoping that society can provide them with a place that is relatively safe and can satisfy their social interaction activities. Combined with the physical characteristics of the old people, the design of activity venues must include natural health factors, which reflects a kind of care and consideration for old people. The event space for the elderly has a reasonable radius to meet the needs of various entertainment activities. It creates a healthy, comfortable, self-cultivation and physical exercise ecological environment for the old people.

## Results and Discussion

### Psychological Characteristics of the Elderly and Their Impact on Public Art Design in the Elderly Rehabilitation Space

Because of changes in the living environment, the social roles and family roles of the old people will also change, which will have a very significant impact on the psychology of the old people. At the same time, the mental state of the old people has changed a lot because of the different nature of the work, the degree of education, the differences in local culture, and the differences in social experience. In the survey, it was found that loneliness, impatience, survival, resentment, attachment, symbiosis, etc. are the common psychological feelings of the old people.

#### Loneliness

Many old people's spouses may die, the circle of life and activities is smaller, and the number of people who can communicate with themselves is less. In addition, the phenomenon of “empty nest” appears. Old people feel lonely when their children are not around. Therefore, most old people are reluctant to communicate, like to be alone, and not willing to participate in group activities. Lonely feelings can have a very negative impact on the lives of the old people.

#### Lost Feeling

Lost feeling is also a situation that the old people often encounter. Old people feel left out and abandoned. This psychological pressure caused some of the old people to change from self-care to dependents. The sense of loss seriously affects the normal living conditions of the old people, which can cause mental tension and frustration in the old people.

#### Inferiority

In the elderly rehabilitation space, the old people will have a feeling of inferiority because of the difference between themselves and others. Especially when the ability is gradually declining and cannot reach a good state, the old people will inevitably have a tendency to feel inferior, and always think in the subconscious that they are no longer able to do so. The inferiority of old people is also a common occurrence. Because of retirement, social status and economic income are gone. At the same time, the physical condition is not as good as before, the elderly need to be taken care of by others. Therefore, they will have a feeling of inferiority.

#### Isolation

Isolation is the most common phenomenon in the old people group, and it is also a very realistic and serious situation. The very important reason for the emergence of these phenomena is that there is no comfort in the life of the old people. Spiritual comfort cannot be satisfied and cannot make an understanding of the spiritual world. Therefore, ideologically, the elderly will create pressure on themselves, and eager for the care and attention of the people around them, especially their relatives and friends. In view of this, in the design of the rehabilitation space for the elderly, it is necessary to solve the problem of the public art design in the space. At the same time, the accurate grasp of the psychological characteristics of the old people is of great help to design and construction of the old facilities and the public art of the elderly rehabilitation space.

Through investigation on some aspects, such as the urban elderly and the rural elderly cannot keep up with the development of society, the old people are the burden of the society and the family, the society is paying more and more attention to the importance of the old people, the old people like to chat with others and make friends, and the old people often feel lonely. The results are shown in Table 1. According to a sample survey, 65.3% of urban old people and 71.8% of rural old people feel that they cannot keep up with the pace of development. 52.5% of urban old people and 61.2% of rural old people consider themselves to be social burdens. It can be seen that the rural old people are far less than the urban old people in their recognition of their own values. Therefore, for the old people in township nursing homes, they will feel that they can't keep up with the pace of development of the times, and they are the burden of family and society. On the emotional issues of the old people, the urban old people who like to chat with others account for 70.9%, and the rural old people account for 76.2%. In terms of making friends, the urban old people who choose to make friends are 63.7%, and the rural old people account for 59.1%. It is also worth noting that the rural old people often feel lonely more often than the urban old people. In short, the old people, especially the rural old people, consider themselves to be the burden of society and cannot keep up with the development of society. Psychological problems are more serious. It is conceivable that this situation will be more serious for the old people of the nursing home, especially in terms of emotional comfort.

**Table 1:** Composition of various statements of the elderly in the surveyed cities and rural areas.

	**City**			**Rural**		
	**Yes**	**Unclear**	**No**	**Yes**	**Unclear**	**No**
Can't keep up with the development of society	65.3%	13.3%	21.4%	71.8%	14.3%	13.9%
Old people are the burden of society	52.5%	16.4%	31.1%	61.2%	19.8%	19.0%
Old people are the burden of the family	46.9%	15.1%	38.0%	67.3%	15.6%	17.1%
The society is increasingly concerned about the importance of the elderly	82.1%	12.3%	5.6%	86.2%	10.5%	3.3%
Older people like to chat with others	70.9%	10.3%	18.8%	76.2%	9.1%	14.7%
Older people like to make friends	63.7%	12.8%	23.5%	59.1%	15.3%	25.6%
Old people often feel lonely	24.9%	11.4%	63.7%	37.6%	16.1%	46.3%

**Figure 3:**
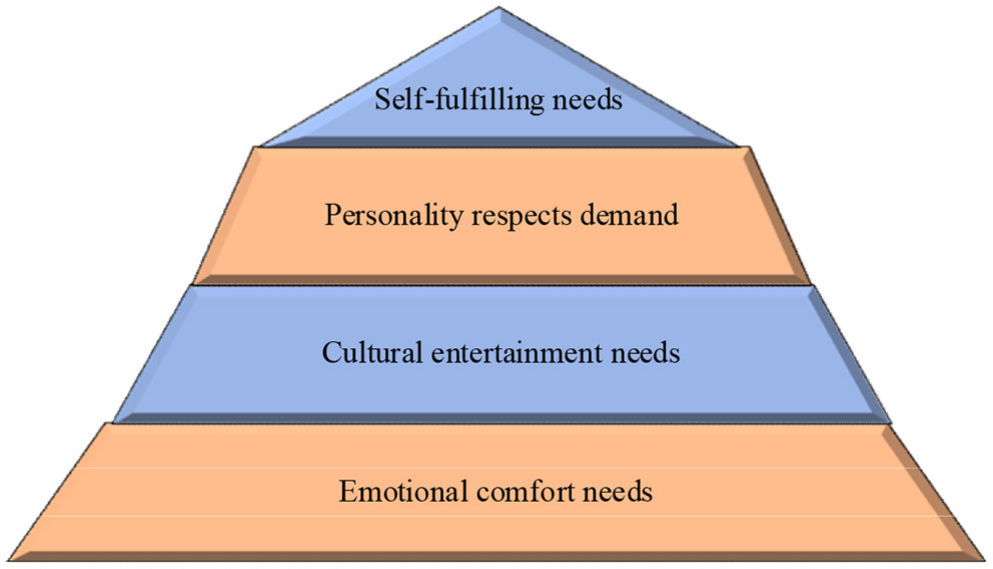
The spiritual needs of the elderly.

Various psychological characteristics were studied. Old people will choose different activities due to different psychological characteristics. Changes in the mental state of the old people will directly affect the design of public art in the old people rehabilitation space. Therefore, the factors influencing this aspect must always be considered in the design. At present, in the elderly rehabilitation space, there are relatively few design studies on the psychological characteristics of the old people. The spiritual needs of the elderly are shown in Figure 3.

#### Safety

The body function of the old people is declining and the movement is inconvenient. The ability to recover from injuries is also weakening. Therefore, security guarantees are important. Environmental design and landscape design in the public space of the elderly must be taken seriously. For example, barrier-free design, emergency call system, emergency system and medical security system are all considerations of old people security requirements.

#### Sense of Belonging

When entering a new living environment and state, the old people need to be recognized by the people and the environment around them to enhance their self-confidence. In this way, these old people can integrate into the collective, build confidence and be accepted and recognized. A sense of belonging is also a sense of responsibility of the old people to the environment and the people around them. Old people who lack a sense of belonging will have a clear lack of responsibility and vice versa.

#### Neighborhood

The neighborhood of the old people is mainly reflected in the social neighborhood and the environmental neighborhood. Social neighborhood means that the old people need to find friends in the new environment who can communicate, exercise and play games with themselves. An environmental neighborhood means that the old people are intimate with the environment of life and are willing to live in this environment. When designing the living space of the old people, the design of the communication environment of the entire event space was improved, and a new neighborhood communication space was created.

#### Family Sense

In the design of the elderly rehabilitation space, in addition to considering the psychological feelings of the old people, the sense of family also needs to be considered. The family atmosphere is created. The old people in the old-age care institutions have left the familiar life field that has lived for decades, and it takes time to adjust the lever for the new living environment. The sense of family in the whole environment depends on the accumulation of time.

### Public Space for the Old People's Convalescence

The public space for elderly rehabilitation is mainly divided by the space range, as shown in Table 2. After a survey, it is found that most people aged 50-70 like to live in public spaces such as communities, squares, activity rooms and outdoors. Therefore, the scope of this design is divided into two parts: indoor space and outdoor space. The indoor public space includes a leisure reading area, display area, reading room, fitness room, yoga room and performance hall. In the outdoor public space, there are rooftop leisure gardens, landscape viewing spaces, and fitness spaces. Indoor public space and outdoor public space together constitute the public space category of this research.

**Table 2:** Analysis of scope of activities.

**Scope of activities**	**Percentage**
Home	16%
Community	20%
Square	32%
Elderly activity room	9%
Outdoor	23%

### Discussion of Results

Through the analysis and discussion of the physiological and psychological characteristics of the old people, the entire survey shows that the physiological functions of the old people are declining, which seriously affects their normal life. Psychologically, old people are also suffering from a variety of social and family role changes. A healthy mindset can help the old people live a happy life, but most of the old people have a hard time accepting the current situation, from the main role of society to retirement. The main difficulty of the old people is to face a new living environment and establish a new personal relationship and emotional space. Therefore, the design of the old people rehabilitation space should analyze the spatial design from the perspective of humanization and emotionality while meeting the basic needs of life. An ecological, natural and humane living environment has been established. In the old people rehabilitation space, public art is designed to fully consider their needs. In the rehabilitation space, older people can spend their old age in happiness. The quality of the living environment is good. Furthermore, connecting people through shared experiences and emotions focuses on connecting people to artworks and connecting them to specific communities, which is consistent with the work of Zebracki et al. (17).

## Conclusion

Today, society is facing an increasingly serious phenomenon of aging, and old people are getting more and more attention. The basic starting point of public art, public environment, and public facilities in the elderly rehabilitation space is to meet the physical and psychological needs of the old people, to facilitate their use of the old people, and to provide a comfortable living environment. At the same time, in order to reduce the psychological pressure of the old people, a healthy, harmonious and happy rehabilitation space was established. However, the current main research task for the construction of old-age care institutions should be based on the psychological and behavioral characteristics of the old people. The society is constantly improving, and the concept of life of the old people in each era is constantly changing and continuing. Therefore, the psychological research on the old people is constantly changing, and the design concept of the elderly rehabilitation space will be constantly changing and updating. Taking the elderly as the research object, their physiological, psychological and behavioral characteristics were studied. Existing geriatric care institutions have been investigated on the spot, which provides relatively intuitive research data for exploring conclusions. The results show that the recuperation space design should be built according to the needs of different old people, which helps the old people to eliminate loneliness. At the same time, to enhance the value of the role, the old people can also make life full of joy and meaning. The shortcoming is that it only analyzes the feasibility of the application of public art experience design for the old people. However, the interactive behavior of the old people's care space is related to each other and affects each other. Therefore, a public space that can give the old people a sense of belonging and comfort should be created as much as possible to meet their psychological and physical needs. Creating a public space environment full of communication and interaction in the rehabilitation space for the old people can not only provide new design ideas and research directions for the public space design of elderly apartments, but also enrich other fields of environmental art design in theory and practice.

## Data Availability Statement

The original contributions presented in the study are included in the article/supplementary material, further inquiries can be directed to the corresponding author.

## Ethics Statement

All subjects gave their informed consent for inclusion before they participated in the study. The study was conducted in accordance with the Declaration of Helsinki and the protocol was approved by the Ethics Committee ofWonkwang University (Project Identification Code 13843/CNA).

## Author Contributions

SZ: writing—original draft preparation and review and editing.

## Funding

This work was supported by the China Postdoctoral Science Foundation.

## Conflict of Interest

The author declares that the research was conducted in the absence of any commercial or financial relationships that could be construed as a potential conflict of interest.

## Publisher's Note

All claims expressed in this article are solely those of the authors and do not necessarily represent those of their affiliated organizations, or those of the publisher, the editors and the reviewers. Any product that may be evaluated in this article, or claim that may be made by its manufacturer, is not guaranteed or endorsed by the publisher.
